# Current Applications of Single-Cell RNA Sequencing in Glioblastoma: A Scoping Review

**DOI:** 10.3390/brainsci15030309

**Published:** 2025-03-14

**Authors:** Edgar G. Ordóñez-Rubiano, Nicolás Rincón-Arias, William J. Shelton, Andres F. Salazar, María Alejandra Sierra, Raphael Bertani, Diego F. Gómez-Amarillo, Fernando Hakim, Matías Baldoncini, César Payán-Gómez, Alba Lucia Cómbita, Sandra C. Ordonez-Rubiano, Rafael Parra-Medina

**Affiliations:** 1 Department of Microbiology, School of Medicine, Universidad Nacional de Colombia, Bogotá 111321, Colombia; 2 Department of Neurosurgery, Fundación Universitaria de Ciencias de la Salud—FUCS, Hospital de San José—Sociedad de Cirugía de Bogotá, Bogotá 110111, Colombia; nrincon@fucsalud.edu.co; 3 Department of Neurosurgery, Fundación Santa Fe de Bogotá, Bogotá 111071, Colombia; d.gomez8@uniandes.edu.co (D.F.G.-A.);; 4 School of Medicine, Universidad de los Andes, Bogotá 110111, Colombia; wj.sheltonc@gmail.com (W.J.S.); andresalazarab@gmail.com (A.F.S.); 5 School of Medicine, Universidad del Rosario, Bogotá 111221, Colombia; mariaaleja.sierra@urosario.edu.co; 6 Division of Neurosurgery, University of São Paulo, São Paulo 01246-904, Brazil; neurocirurgia@rbertani.com; 7 Laboratory of Microsurgical Neuroanatomy, Second Chair of Gross Anatomy, School of Medicine, University of Buenos Aires, Buenos Aires B1430, Argentina; drbaldoncinimatias@gmail.com; 8 Dirección Académica, Universidad Nacional de Colombia, Sede de La Paz, Cesar 202017, Colombia; 9 Grupo de Investigación Traslacional en Oncología, Instituto Nacional de Cancerología, Bogotá 111321, Colombia; 10 Department of Chemistry, School of Humanities and Sciences, Stanford University, Stanford, CA 94305, USA; sc.ordonezr@gmail.com; 11 Department of Pathology, Instituto Nacional de Cancerología, Bogotá 111511, Colombia; rparram@cancer.gov.co; 12 Research Institute, Fundación Universitaria de Ciencias de la Salud—FUCS, Hospital de San José—Sociedad de Cirugía de Bogotá, Bogotá 111711, Colombia

**Keywords:** single-cell RNAseq, glioblastomas, gliomas, next-generation sequencing

## Abstract

**Background and Objective:** The discovery of novel molecular biomarkers via next-generation sequencing technologies has revolutionized how glioblastomas (GBMs) are classified nowadays. This has resulted in more precise diagnostic, prognostic, and therapeutic approaches to address this malignancy. The present work examines the applications of single-cell RNA sequencing (scRNA-seq) in GBM, focusing on its potential to address tumor complexity and therapeutic resistance and improve patient outcomes. **Methods:** A scoping review of original studies published between 2009 and 2024 was conducted using the PUBMED and EMBASE databases. Studies in English or Spanish related to single-cell analysis and GBM were included. **Key Findings:** The database search yielded 453 publications. Themes related to scRNA-seq applied for the diagnosis, prognosis, treatment, and understanding of the cancer biology of GBM were used as criteria for article selection. Of the 24 studies that were included in the review, 11 focused on the tumor microenvironment and cell subpopulations in GBM samples, 5 investigated the use of sequencing to elucidate the GBM cancer biology, 3 examined disease prognosis using sequencing models, 3 applied translational research through scRNA-seq, and 2 addressed treatment-related problems in GBM elucidated by scRNA-seq. **Conclusions:** This scoping review explored the various clinical applications of scRNA-seq technologies in approaching GBM. The findings highlight the utility of this technology in unraveling the complex cellular and immune landscapes of GBM, paving the way for improved diagnosis and personalized treatments. This cutting-edge approach might strengthen treatment strategies against tumor progression and recurrence, setting the stage for multi-targeted interventions that could significantly improve outcomes for patients with aggressive, treatment-resistant GBMs.

## 1. Introduction

Integrating molecular biomarkers into diagnosing and classifying central nervous system (CNS) tumors has significantly advanced our understanding of these diseases by revealing unique molecular characteristics that underscore tumor diversity. This progress has enabled a more precise diagnosis, treatment, and prognosis. Traditionally, CNS tumor classification depended solely on histologic analyses of tissue samples [1]. However, the complexity and aggressive behavior of many CNS tumors have necessitated new classification methods that address the high mortality and recurrence rates often seen in malignant cases [2]. Glioblastoma (GBM) is a prime example. While relatively rare, with an incidence rate of 0.58 per 100,000 from 2016 to 2020, as documented by the Central Brain Tumor Registry of the United States, GBM remains the most frequently diagnosed malignant brain tumor [3]. Despite multimodal treatments, including maximal safe resection followed by adjuvant chemoradiotherapy, GBM has a poor prognosis, with a median overall survival of approximately 14 months and a 5-year survival rate of only 5% [4].

With the advancements in sequencing technologies, detailed tumor characterization based on individual genetic composition is now possible. Multi-omics analyses—encompassing genomics, transcriptomics, and epigenomics—have become increasingly valuable for classifying and identifying molecular subtypes of CNS tumors. Part of the complexity of these tumors, and especially GBM, falls on the intrinsic biological complexity of cancer. Tumor heterogeneity plays a fundamental role in the progression and resistance of the disease by enabling malignant cells to morph into different cellular states in response to the determined stimuli [5], such as the tumor microenvironment (TME) [6]. Therefore, applying novel sequencing technologies, such as single-cell RNA sequencing (scRNA-seq), which features the analysis of the transcriptome of individual cells, opens new avenues for addressing diverse, aggressive, and highly resistant tumors like GBMs. In this scoping review, we examine the applications of scRNA-seq regarding the diagnosis, treatment, and prognosis of GBM.

## 2. Materials and Methods

A scoping review was conducted to gather the most up-to-date information for this current review. Emphasis was placed on literature that examined the various applications of scRNA-seq analysis in GBM. Specifically, the focus was on articles exploring scRNA-seq applications in the diagnostic, prognostic, and therapeutic contexts of GBM. Additional attention was given to articles involving the translational applications of scRNA-seq in GBM using in vivo and in vitro models for an improved understanding of cancer biology. Original studies written in English or Spanish and indexed in PUBMED and EMBASE from 2009 to 2024 were included. A total of 453 articles were screened, which resulted from the following search terms: ((“Single-Cell Gene Expression Analysis” [MAJR]) OR “Single-Cell Analysis” [MeSH]) OR “Sequence Analysis, RNA” [MeSH] OR “RNA-Seq” [MeSH] OR “Gene Expression Profiling” [MAJR] AND (glioblastoma [MeSH Terms]). Articles were selected and filtered based on their abstracts. Articles not related to scRNA-seq applications in GBM were excluded. Twenty-four articles were summarized, and the main findings (cell populations and relevant genes) were reported in Table 1. The current roles of scRNA-seq in glioblastomas are summarized in Table 2.

## 3. Review

### 3.1. General Aspects of scRNA-seq

Medicine is undergoing a profound transformation as it embraces the molecular era, which has become particularly relevant in recent years, especially within oncology. This shift aims to deepen our understanding of the cellular makeup of diseases and their interactions with their microenvironment, enabling a more precise approach to identifying the origin of disease. Such advancements facilitate more accurate, less invasive diagnostics and open the door to identifying new therapeutic targets, ultimately paving the way for more tailored and effective treatments [31]. In this scoping review, 453 articles were screened, of which 24 were selected and summarized in Table 1. The major subtopics identified were as follows: the tumor microenvironment (n = 11), the cancer biology of GBM (n = 5), prognosis through scRNA-seq models (n = 3), translational research (n = 3), and treatment in GBM (n = 2).

Analyzing individual cells through comprehensive molecular approaches has become crucial for moving forward our understanding of disease and improving our insights into whole tissues and organs, particularly through detailed information on intrinsic and microenvironmental interactions [32]. The transcriptome, in particular, is integral to cell identity, correlating with specific cellular phenotypes and changes. Therefore, a single-cell transcriptomic analysis via scRNA-seq provides unique insights into the cell-to-cell variation.

**Figure 1:**
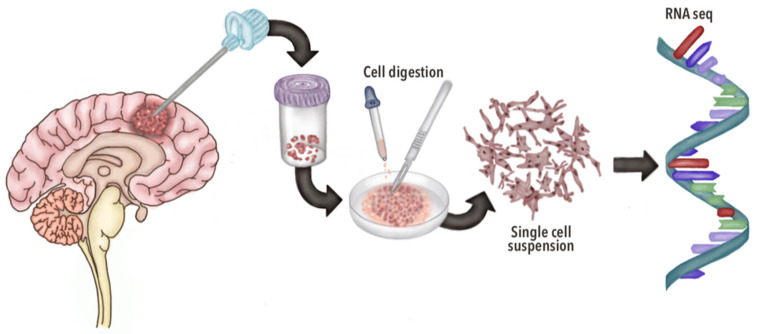
Model for exploring the cellular states of glioblastoma and their genetic and microenvironmental determinants through scRNA-seq: GBM tissue is sampled, followed by enzymatic cell digestion to obtain a single-cell suspension. Cells are then subjected to RNA sequencing to reveal gene expression patterns. This approach highlights (1) inter-patient heterogeneity, showing fragmented clustering and subtype mixtures, and (2) intratumoral heterogeneity across (a) spatial distribution, (b) oncological and non-oncological cellular heterogeneity, and (c) oncological and non-oncological cellular organization.

**Figure 2:**
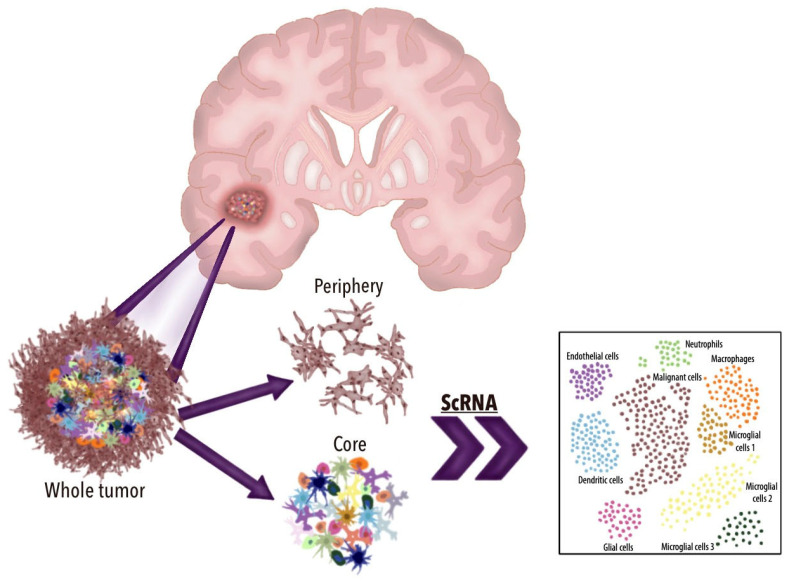
Illustration depicting the use of scRNA-seq in GBM: scRNA-seq technologies enhance the understanding of GBM by revealing individual cell populations within the tumor. This illustration demonstrates how generating a uniform manifold approximation (UMAP) through scRNA-seq can highlight key features of GBM, such as intratumoral heterogeneity and the tumor microenvironment.

**Figure 3:**
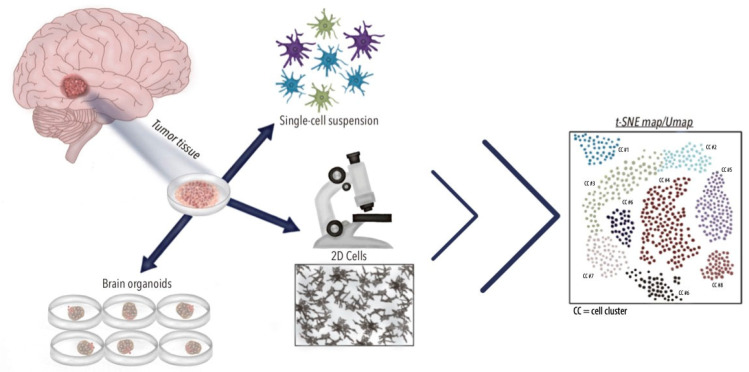
Applications of Single-Cell RNA Sequencing in GBM. Single-cell RNA sequencing technologies in glioblastoma facilitate various downstream analyses using surgical samples. By dissociating tumor cells into single cells, researchers can conduct both in vivo and in vitro studies to investigate critical components of cancer biology such as intratumor heterogeneity. Comparing in vivo and in vitro studies using Sc-RNA-seq technologies, such as the application of brain organoids and 2D cell cultures, provides valuable insights into the tumorigenesis of GBM. Uniform manifold approximation through scRNA-seq: UMAP.

The scRNA-seq technique begins with the precise isolation of individual cells from sources such as tissue samples, dissociated cell suspensions, or cultures. The RNA from the cells is then isolated, converted into complementary DNA (cDNA), and subsequently subjected to the high-throughput sequencing of cDNA libraries [33]. With the sequencing data and gene expression profiling, scRNA-seq reveals previously unrecognized transcriptional similarities and differences within bulk populations once thought to be molecularly uniform (Figure 1, Figure 2 and Figure 3). This level of precision has greatly enhanced our understanding and characterization of complex diseases, such as CNS tumors, allowing for a more accurate and nuanced classification. Through scRNA-seq, researchers can now identify rare cell types and subpopulations, providing critical insights that were previously out of reach [32].

The plasticity of GBM cells further complicates treatment strategies. Single-cell-derived clones from the same patient can exhibit vastly different proliferative, differentiative, and drug-response profiles, emphasizing the need for multi-region biopsies to capture the full spectrum of tumor heterogeneity. For example, recent scRNA-seq analyses of patients with multifocal GBM (arises from a common precursor and undergoes parallel evolution) have identified a natural evolution signature (NES) characterized by the activity of genes such as HIF1A, FOSL2, and ANXA1, which increases throughout tumor evolution. This NES provides a framework for understanding the molecular drivers of heterogeneity and could inform the development of targeted therapies [34].

The integration of the scRNA-seq and phylogenetic analyses has significantly advanced our understanding of GBM heterogeneity, evolution, and therapeutic resistance. These findings underscore the need for personalized, multi-targeted approaches that account for the dynamic and diverse nature of GBM. By identifying universal markers, shared invasion pathways, and novel therapeutic targets, recent research has paved the way for more effective treatments and improved patient outcomes in this devastating disease which will be discussed further in this review.

### 3.2. In Vitro and In Vivo Applications of scRNA-seq in GBM

ScRNA-seq has been widely employed in both in vitro and in vivo studies of GBM. As has been stated, using this technology addresses disease understanding with fundamental tools such as gene expression profiling. Recently, the implementation of three-dimensional cell culture systems, such as brain organoids (Figure 3), has gained popularity, given their higher architectural resemblance with in vivo tissues compared to monolayer or 2D cultures [35]. Three-dimensional cell cultures are advantageous tools as they can recapitulate the cellular heterogeneity found in GBM [10,30,36]. Similarly, patient-derived explants have been shown to mimic primary tumors by retaining similar transcriptomic profiles to that of the primary tumor [10]. A study by LeBlanc et al. analyzed bulk exome and single-cell genomes and transcriptomes from primary GBMs with matched patient-derived explants and gliomasphere lines. A transcriptomic analysis by scRNA-seq demonstrated the similarity of patient-derived explants in retaining similar genetic characteristics and transcriptional heterogeneity to that of primary tumors [10].

The use of scRNA-seq in in vivo studies also provides valuable information regarding the immune response toward tumor growth. Identifying the responses and the cellular components present in GBM is critical for establishing pivotal mechanisms of the disease. For example, a mouse model of GBM examined the immune landscape of GBMs by performing scRNA-seq on both newly diagnosed GBM patient samples and orthotopic GL261 tumors in mice [37]. The use of this technology identified a diverse immune population in the different subsets of cells found, such as dendritic cells and tumor-associated macrophages (TAMs) [37]. A transcriptomic analysis of individual cells highlights the importance of these technologies for the understanding of the behavior and response of GBM to its immune microenvironment [38]. scRNA-seq provides information on how similar or different the immune landscapes of patients and mice tumors are, enhancing the potential information extracted from scRNA-seq in mouse studies of GBM.

### 3.3. TME Dynamics in GBM and scRNA-seq Applications

The TME of GBM is a complex and dynamic ecosystem that plays a critical role in tumor progression, therapy resistance, and immune evasion. It can be broadly divided, as described by Hambardzumyan et al. [39], into three distinct regions: the hypoxic tumor region at the core, the perivascular tumor region, and the vascular-invasive tumor region, each characterized by unique cellular and molecular composition. The advent of scRNA-seq has revolutionized our understanding of the TME by enabling the detailed characterization of its diverse cell populations and their functional states. These insights are required to better understand the multiple cellular interactions that are particularly relevant in the context of immunotherapy, where more understanding is required to further delineate the multiple immunosuppressive mechanisms of GBM that lead to the limited efficacy of the immune checkpoint blockade not seen in other cancer types [39,40].

The GBM TME is characterized by a highly immunosuppressive landscape, primarily driven by the abundance of myeloid cells and regulatory T cells. Myeloid cells constitute approximately 45% of the total TME and encompass a diverse array of cell types, including macrophages, microglia, granulocytes, monocytes, and myeloid-derived suppressor cells (MDSCs). Notably, sex-specific differences have been observed in the myeloid compartment, with female GBM patients exhibiting a higher proportion of myeloid cells compared to males [41,42]. This finding highlights the potential influence of sex on the TME composition and therapeutic responses, also highlighting the interpatient heterogeneity of GBM. Another key component of the GBM TME are TAMs, which exhibit remarkable interindividual differences. While in vitro studies classify macrophages into pro-inflammatory M1 and immunosuppressive M2 subtypes, in vivo TAMs often co-express markers of both states. scRNA-seq has revealed diverse TAM subpopulations, including SPP1-expressing, IFN-activated, proliferating, inflammatory, and MHCII-high macrophages. SPP1-expressing TAMs have been particularly implicated in promoting glioma cell survival and angiogenesis, making them another potential therapeutic target [43]. Additionally, GBM cells can induce the expression of surface markers on TAMs such as CD78 that lead to their conversion into immunosuppressive participants by producing adenosine that further inhibits CD8^+^ T-cell activity. Another immunosuppressive TAM subset has been shown to express the MARCO receptor, which is associated with poor clinical outcomes [37,40,44].

In addition to macrophages, both MDSCs and T cells play an important role in the TME of GBM, having important immunosuppressive functions and contributing to tumor progression. MDSCs support glioma cells, by creating an immunosuppressive environment within the tumor microenvironment, inhibiting T-cell proliferation and exhibiting increased epigenetic immunoediting, hence facilitating immune evasion [45]. A particular polymorphonuclear subtype of MDSCs, which is absent in normal brain tissue is more prevalent in IDH1 wild-type tumors. This specific subtype is involved in epigenetic reprogramming, hence explaining the poorer prognosis of these patients compared to IDH1 mutant cases [16]. Despite their importance, distinguishing MDSCs in scRNA-seq datasets remains challenging due to the overlapping marker expression with other myeloid populations. Advances in scRNA-seq resolution and complementary techniques are needed to better characterize MDSCs and develop targeted therapies.

scRNA-seq has allowed the identification of four main T-cell populations: CD8^+^ T cells, CD4^+^ conventional T cells, CD4^+^ regulatory T cells (Tregs), and cycling T cells [11]. Tregs and exhausted T cells are abundant in the GBM TME and contribute to immunosuppression through IL-10 secretion [46]. In addition to this, the myeloid cell interplay with T cells has been shown to be an important mechanism via the IL-10 secretion from macrophages, involved in further T-cell exhaustion [47].

Even though B cells are relatively scarce in GBM, they have proven to play a role in tumor progression and immune regulation. GBM cells can convert B cells into immunosuppressive regulatory B cells [48]. Other important participants of TME in GBM are cancer-associated fibroblasts (that promote tumor progression) and endothelial cells that actively modify the blood–brain barrier (BBB). scRNA-seq has identified distinct endothelial cell clusters in GBM that could potentially become therapeutic targets and further improve the significant obstacle of drug delivery in GBM [15].

The application of scRNA-seq has provided insights into the cellular and molecular complexity of the GBM TME. By delineating the heterogeneity of immune and stromal cell populations, studies have identified key players in tumor progression, immune evasion, and therapy resistance. As scRNA-seq technologies continue to advance, they will further illuminate the intricate interactions within the GBM TME, paving the way for more effective and personalized treatments.

### 3.4. Potential Diagnostic Applications of scRNA-seq in GBM

GBM is an exceptionally heterogeneous tumor, presenting significant challenges for characterization. This complexity stems from the diverse range of cell types coexisting within the TME, including malignant cells, endothelial cells, fibroblasts, and a variety of immune cells. Each of these cellular components contributes uniquely to the tumor’s behavior, progression, and resistance to treatment, highlighting the need for advanced techniques to fully understand GBM’s intricate cellular landscape [12]. Genetic, epigenetic, and TME factors profoundly influence the composition and behavior of neoplastic cells in GBM. Single-cell genomics, especially scRNA-seq, has become invaluable for dissecting the complex transcriptomic dynamics within the TME, revolutionizing our understanding of GBM [28,49,50]. This precision enables researchers to identify key genes that define cancer cell subtypes and drive tumor behavior, providing critical insights for targeted therapies [51].

Tumor heterogeneity in GBM is pronounced not only across different stages of tumor development but also between genders and age groups. The invasive and metastatic potential of GBM cells further amplifies this variability. Even within a single tumor, cells may harbor unique mutations, leading to diverse phenotypic and epigenetic landscapes. Despite shared genetic characteristics between tumoral cells, including the amplification of chromosome 7 and deletion of chromosome 10, individual patients with GBM demonstrate a diverse array of genomic aberrations. This variability contributes to the distinctiveness of each tumor [21,52]. This complexity poses diagnostic challenges that scRNA-seq is well-equipped to address, providing a precise cellular and genetic diagnosis tailored to the individual characteristics of each case [49,53,54].

Recent advances in scRNA-seq have provided insights into the cellular and molecular diversity of GBM. For example, SOX2 has emerged as a potential marker for transformed glioma cells, as its high expression is consistently observed in high-grade glioma cells [55]. Additionally, studies mapping the phylogenetic evolution of GBM have revealed that mutations in EGFR tend to accumulate at later stages of tumor development, while alterations in PI3KCA occur earlier [27]. These findings highlight the dynamic nature of GBM evolution and suggest that therapeutic efficacy may depend on the genetic similarity between the tumor and the targeted intervention [56]. In this way, an accurate diagnosis throughout the evolution of GBM in time (embracing all spatiotemporal genomic architecture) would also give hope for a perfect timing in specific targeting [27]. However, there is scarce information regarding the GBM spatiotemporal genomic information acquired from scRNA-seq.

Understanding the geographical distribution of tumors is essential for elucidating GBM propagation behavior regarding dissemination and immune infiltration [27]. It is paramount to analyze biopsies from various locations and time points to characterize the spatiotemporal genomic architecture of GBM. Some findings have revealed that multifocal tumors exhibited a greater genetic diversity than adjacent tumors, highlighting significant spatial genetic heterogeneity [27]. Similarly, Yu et al. demonstrated that a single biopsy and RNA-seq of bulk tissue failed to capture the intratumoral and TME heterogeneity, as individual GBM cells displayed distinct subtypes that varied dramatically across different regions of the same tumor [25].

The diffuse nature of GBM has led to the hypothesis that the recurrence of the disease following tumor resection may be attributed to the presence of residual long-distance migratory oncological cells [57,58]. Darmanis et al. performed separate biopsies of the tumor core and periphery, followed by scRNA-seq [20]. Their analysis revealed that the tumor core exhibited a higher expression of adhesion- and hypoxia-related genes compared to the tumor margin. They also found a smaller percentage of proliferating cancer cells in the infiltrating fraction, with a greater abundance of these cells in the tumor core [20]. This finding supports previous studies, indicating that hypoxia enhances glioma stem cell expansion through HIF-1α expression [52]. Notably, infiltrating tumoral cells obtained from tumor periphery samples exhibited a converging mechanism of dissemination [20].

Single-cell genomics from patient-derived tumor specimens resected during routine disease management or research biopsies holds great promise for advancing discoveries and improving therapy deployment in GBM [19,54]. While the underlying mechanisms of the epithelial-to-mesenchymal transition in recurrent GBM remain unclear, several factors related to standard therapies may contribute. These include variations in cellular division rates within mesenchymal and non-mesenchymal populations, individual cells transitioning to a mesenchymal phenotype, quiescent mesenchymal cells demonstrating preferential resistance to standard treatments, and genetic alterations favoring the mesenchymal state [59,60]. Although RNA velocity analyses indicate that the contribution of phenotypic shifts is modest, the timing of the sample collection complicates this assessment. Samples taken months after treatment pressure has been removed suggest that treatment-induced phenotypic shifts may significantly influence the later prevalence of mesenchymal cells [61]. The findings of Wang et al., along with prior studies, support the existence of quiescent, stem-like cells with mesenchymal characteristics that are resistant to ionizing radiation and temozolomide [62]. These cells can re-enter the cell cycle after therapy, potentially driving disease recurrence [62]. scRNA-seq has been invaluable in revealing these dynamic changes, enhancing our understanding of cellular heterogeneity and the transcriptional profiles of individual cells, thereby informing targeted therapies and improving prognostic strategies for GBM patients [62].

Other potential diagnostic roles of scRNA-seq are to identify different GBM metabolic subtypes. Garofano et al. were able to use scRNA-seq to identify a potential GBM subtype with therapeutic vulnerabilities [63]. The mitochondrial subtype of GBM stands out for its dependence on oxidative phosphorylation for energy production, in contrast to the glycolytic/plurimetabolic subtype, which relies on aerobic glycolysis as well as amino acid and lipid metabolism [63]. Clinically, the mitochondrial subtype is linked to a more favorable prognosis and demonstrates a heightened sensitivity to oxidative phosphorylation inhibitors. A defining genetic feature of this subtype is the deletion of the glucose-proton symporter SLC45A1 [63]. Its reintroduction induces cellular acidification and diminishes the fitness of mitochondrial glioma cells.

Many of the networks inferred to be derived from scRNA-seq represent well-characterized pathways associated with GBM, including those involved in the inflammatory response (e.g., type II interferon, and interleukin-1 to -4) [62,64], immune cell chemotaxis (e.g., CCL/CXCL, and colony-stimulating factors) [65], and angiogenesis (e.g., platelet-derived growth factor and vascular endothelial growth factor (VEGF)) [66,67]. These pathways remain active from the initial stages of the disease to recurrence. However, specific ones were notably upregulated in recurrent tumors, particularly within mesenchymal cells. Importantly, during recurrence, mesenchymal cells exhibited the expression of receptors associated with the WNT, NRG, NGF, and IGF signaling pathways, thereby providing a novel set of markers for classifying the more aggressive recurrent forms of GBM [62].

As the number of studies employing scRNA-seq to analyze GBM patient samples increases, the clearer the transcriptomic landscape of GBM in different contexts will become. This will result in additional defined tumor markers for a more accurate staging and diagnosis of GBM.

### 3.5. Prognostic Applications of scRNA-seq in GBM

Several studies have examined the possibility of implementing scRNA-seq for establishing prognostic models in GBM. For example, Lai et al. [24] constructed a prognostic model for survival prediction in patients with GBM, utilizing two public datasets containing scRNA-seq and bulk RNA-sequencing data. In this study, based on 43 differentially expressed genes that were correlated with overall survival among both datasets, a “five-gene-based risk score prognostic model” was built [24]. This model, which was externally validated, demonstrated significance in the overall survival between high- and low-risk groups, showing a worse prognosis in the high-risk group [24].

Similarly, a study carried out by Wu et al. analyzed six tumor tissue samples from six patients with GBM using scRNA-seq (three were catalogued as primary and three as recurrent). In this study, the single-cell transcriptomes were retrieved and used from 30,126 cells for unbiased clustering, resulting in the identification of 11 major cell types and 16 subclusters present in the tumor population [7]. The analysis of each subcluster by a differential gene expression analysis identified markers of poor prognosis, such as certain enriched-signaling pathways and cells with a high expression of proliferation-related proteins, all associated with a poor prognosis in patients with GBM [7,68,69,70].

The use of scRNA-seq technologies enables the identification of novel biomarkers useful for predicting disease aggressiveness. By examining key molecular processes, such as cancer cell resistance to programmed cell death, a more accurate prognosis can be established. For example, Zheng et al. utilized scRNA-seq for the identification of necroptotic genes in 169 GBM patients [9]. The use of this technology established a prognosis risk model involving relevant necroptosis-related genes, such as NDUFB2, which might be correlated with poor outcomes in GBM [9].

Understanding the biological mechanisms that give rise to GBM is fundamental for advancing the comprehension of this disease. Utilizing scRNA-seq technologies makes it possible to understand the molecular characteristics of cancer cells that contribute to a poor prognosis by highlighting the essential steps and processes that contribute to the formation and aggressiveness of GBM.

### 3.6. Therapeutic Applications of scRNA-seq in GBM

#### 3.6.1. Precision Medicine

After The Cancer Genome Atlas Consortium (TCGA) provided the numbers for recurrent genomic aberrations in GBM, data analysis efforts by Verhaak et al. in 2010 allowed for the classification of GBM into neural, pro-neural, classical, and mesenchymal [71]. While this clustering proved to encompass all the data collected, the intratumoral diversity of GBM remained to be addressed until single-cell next-generation sequencing techniques were developed.

Averaging data from tumor samples obscures individual cells’ unique characteristics and biomarkers that may dictate the therapeutic response. Recently, cancer therapy has shifted from traditional systemic treatments on population-founded evidence to rather more personalized approaches [52]. Precision medicine aims to identify diagnostic, potentially therapeutic, and prognostic molecular features in tumors, which is particularly challenging in GBM due to its inherent heterogeneity. Addressing the cellular diversity in GBM, Wang et al. employed scRNA-seq data to classify cells as single glioma cells or tumor-associated host cells and identified genes unique to the single glioma cells. Additionally, higher levels of M2 macrophages and a poor response to ionizing radiotherapy were correlated, and so were the frequency of CD8^+^ lymphocytes and hypermutations. Adequate TME individual cell characterization promises to be key in developing precise medicine for patient care.

Intratumoral cell classification has also been reported by Neftel et al., who developed a model for GBM portrayal based on cell lineage tracing, genetics, and the microenvironment by integrating the scRNA-seq of 28 tumor samples with a bulk RNA-seq analysis of TCGA data and experimental models [19]. Four main cellular states were identified within the different malignant tumor cells, neural-progenitor-like, oligodendrocyte-progenitor-like, astrocyte-like, and mesenchymal-like, which exist in different proportions in each tumor and are characterized by genetic aberrations in *CDK4*, *PDGFRA*, *EGFR*, and *NF1*. scRNA-seq has allowed for GBM characterization at a resolution that traditional bulk tissue molecular analysis cannot achieve.

Recent studies employing scRNA-seq have also offered profound insights into the cellular and molecular alterations of the BBB in GBM. For example, Xie et al. identified distinct endothelial cell phenotypes within GBM, each exhibiting varying degrees of BBB disruption and correlating with specific tumor regions [15]. This endothelial heterogeneity suggests that BBB permeability could be strategically modulated by targeting key molecular pathways, potentially improving drug delivery to the tumor [15].

Single-cell characterization has increasingly become more important in cancer research, particularly in cancers with profound intratumoral heterogeneity, such as GBM. This heterogeneity impedes adequate diagnosis and staging and has hindered drug discovery efforts, as exemplified by Rindopepimut, a vaccine designed to target the EGFRvIII mutant that failed to prove effective in phase III of clinical trials. Despite its initial promise in recognizing and targeting the mutant EGFR protein, the vaccine’s inability to achieve significant clinical benefits underscores the complexity of GBM biology and the limitations of single-target approaches [72]. The heterogeneity of GBM is reflected in the diverse copy number alterations in key genes that drive the emergence of distinct cell lineages within the same TME, as well as the coexistence of multiple subtypes within a single tumor [72].

#### 3.6.2. Chemotherapy

The use of molecular markers to predict the response of GBM to chemotherapy is a relevant research topic. In clinical practice, the identification of markers using various techniques is well-documented, including the MGMT methylation status, detection of IDH-1 and IDH-2 mutations, Ki67 labeling index, EGFR amplification, and PTEN deletion, among others [73].

The role of scRNA-seq in GBM sample analysis can be regarded as an additional value as it is possible to adequately identify a plethora of biomarkers and provide more specific information than conventional diagnostic methods. For example, Wu et al. described the overall VEGF overexpression across nearly all clusters in recurrent GBM [7]. The reshaping of the TME in recurrent GBM leads to increased levels of the VEGFA isoform, which stimulates angiogenesis and tumor growth [7]. Previous studies have shown a negative correlation between serum VEGFA levels and the effectiveness of bevacizumab treatment, a VEGF inhibitor [74]. As a result of recurrent GBM samples via scRNA-seq, VEGFA may be categorized as a biomarker of recurrent GBM that shows a poor response to bevacizumab. Additionally, higher levels of genes associated with the MGMT signaling pathway correlated with a poor prognosis in recurrent GBM [7]. This aligns with MGMT being a well-established biomarker of an inadequate response to temozolomide, demonstrating the applicability of scRNA-seq in providing a detailed analysis of biomarkers of response to chemotherapy.

#### 3.6.3. Immunotherapy

While common immunotherapy-success indicator biomarkers in cancer include mutational burden and checkpoint ligand expression, there is a significant lack of validated biomarkers characterized for GBM. scRNA-seq offers the potential to discover new biomarkers and inform GBM immunotherapy by providing detailed transcriptomic outlines of the immune microenvironment. A promising direction for future research is proposed by Muller et al. through targeting immunosuppression-associated TAMs derived from peripheral blood rather than brain-resident microglia to enhance the host immune response against GBM [22]. By elucidating this heterogeneity, scRNA-seq can help identify tumor antigens expressed by the majority of clones within a tumor, thereby guiding the selection of appropriate immunotherapies [75,76,77].

#### 3.6.4. Radiotherapy

It is well-established that numerous recurrent cases of GBM originate from regions located beyond the resected contrast-enhancing portion of the tumor [26,78]. This recurrence, which persists despite adjuvant radiation and chemotherapy, underscores the necessity of re-evaluating our understanding of single cells within the peritumoral brain zone. Research has shown that GBMs contain stem cells with a marked resistance to radiation [79]. Some of them evade or escape radiotherapy-induced cellular senescence [80]. Although the insights from these studies are not yet clinically actionable, a more profound understanding of single-cell dynamics at the tumor margins could ultimately pave the way for more effective and targeted radiotherapy strategies in this challenging clinical context.

#### 3.6.5. Mechanisms of Resistance

The application of scRNA-seq has also shed light on relapse mechanisms in GBM. For example, mutations in the RAS/GEF GTP-dependent signaling pathway have been identified in relapsed GBM but not in primary tumors, providing insights into the molecular changes underlying treatment resistance. Additionally, studies on H3K27m-glioma have revealed that these tumors are predominantly composed of oligodendrocyte precursor cell (OPC)-like populations, which exhibit a high proliferative capacity and depend on PDGFRA signaling. Targeting these populations could offer a novel therapeutic strategy for this aggressive subtype [81]. Furthermore, scRNA-seq studies have identified key metabolic pathways, such as those involving ELOVL2, which promote GBM tumorigenicity. Knockdown of ELOVL2 has been shown to inhibit tumor growth, highlighting its potential as a therapeutic target [82]. Similarly, as also stated by Wang et al. [81], the discovery of RAD51AP1 as an oncogene in EGFRvIII-driven GBM opens new avenues for combination therapies, such as temozolomide with RAD51AP1 inhibition, which could enhance treatment efficacy and improve patient outcomes.

#### 3.6.6. Technical Limitations and Challenges of scRNA-seq in GBM Research

scRNA-seq has undeniably transformed our understanding of GBM heterogeneity and TME dynamics. However, its application is not without significant technical and analytical challenges that must be critically addressed to ensure robust and reproducible findings. A primary concern lies in the pervasive issue of batch effects that can lead to biological signal confounding, impeding accurate conclusions about cellular states, gene expression patterns, and cellular interactions. Differences in experimental conditions (e.g., sequencing platforms, and sample processing times) can introduce systematic variations in data. This issue is especially critical in GBM, where distinguishing subtle differences between tumor subclones or immune cell states is essential. Such variations may overestimate or distort the true biological behavior [83,84]. Another major limitation is the inherent technical noise in scRNA-seq data from amplification biases and dropout events, where mRNA molecules fail to be captured or amplified. These dropout events, especially seen in low-abundance transcripts, potentially mask rare but functionally critical cell populations and further complicate the interpretation of gene expression profiles. This is especially relevant in GBM, where diverse tumor cells and immune infiltrates in the TME, along with amplification biases, hinder the identification of reliable biomarkers and therapeutic targets [85].

Integrating data from multiple sources can be computationally demanding and can potentially lead to a loss of information if not handled properly. Additionally, integrating scRNA-seq findings across different studies is hampered by differences in cell isolation methods, sequencing depth, or platform-specific biases [86]. These challenges are particularly critical in GBM research where multimodal research approaches are required to further comprehend the TME. Beyond technical limitations, practical and ethical considerations also loom large. The high cost of scRNA-seq and the expertise required for data analysis restrict its accessibility, particularly in low-income settings.

Overcoming these challenges will demand innovations in both the experimental and computational domains. By confronting these technical and ethical hurdles, the field can potentially unlock the full potential of scRNA-seq to dissect GBM’s complexity, identify novel therapeutic susceptibilities, and, ultimately, improve outcomes for patients by posing new opportunities for personalized treatments.

### 3.7. Future Perspectives

By investigating cells at the single-cell level, we can explore the intricate interactions between intrinsic GBM cellular processes and external factors. To date, most scRNA-seq has been performed on freshly isolated samples, but advancements now enable the study of transcriptomics in fixed or cryopreserved tissue samples [87]. Emerging technologies aim to minimize transcriptome perturbations caused by cellular dissociation techniques, which will enhance the accuracy of these analyses. Additionally, improvements in cost-effectiveness will make scRNA-seq more affordable on a per-cell basis, allowing researchers to study not only hundreds but also millions or even billions of cells, thereby facilitating its application in clinical settings. By creating more extensive cDNA libraries, scRNA-seq datasets can be constructed and analyzed to provide deeper insights into cellular behavior, interactions, and lineages. Bioinformatics will undoubtedly face challenges in developing user-friendly interfaces to organize and extract biological information from these vast datasets [31]. In the coming years, scRNA-seq could become a valuable tool for identifying rare malignant and chemotherapy-resistant GBM cells, ultimately guiding treatment decisions and uncovering new therapeutic targets.

With the rise of immunotherapy in cancer treatment, scRNA-seq has the potential to provide valuable insights into the immunologic response. Furthermore, in today’s era of molecular clinical diagnostics, where liquid biopsies are gaining prominence, scRNA-seq holds great promise for diagnosing tumors and monitoring disease progression and treatment response [88]. As scRNA-seq technologies continue to evolve and become more accessible, this powerful technique is well-positioned to move from specialized research laboratories into standard use by both basic scientists and clinicians.

## 4. Limitations

A particular limitation of this study is related to the current definition of GBM. According to the 2021 classification of CNS tumors, GBM is characterized as an adult-type glioma presenting as an IDH wild-type tumor. However, this scoping review included studies published before 2021, which have previously classified GBMs as grade 4 astrocytomas (IDH-mutant). Although the scope of this article is not intended to draw any conclusions, we find it relevant to clarify this aspect.

## 5. Conclusions

scRNA-seq has revolutionized GBM diagnostics by identifying diverse tumor cell populations, mapping spatiotemporal genomic architecture, and uncovering immune escape mechanisms. Additionally, scRNA-seq facilitates more accurate diagnoses and innovative therapeutic strategies, enabling tailored treatments that address tumor progression, treatment resistance, and recurrence while enhancing prognostic precision. Ultimately, scRNA-seq paves the way for personalized, multi-targeted interventions that improve management and outcomes for patients with GBMs.

## Figures and Tables

**Table 1:** Characteristics of studies about microenvironmental determinants in GBM through scRNA-seq.

Author and Year	Cells Identified	Most Important Genes Identified	Conclusions
Wu et al. [7] 2023	10,144 cells from primary GBM and 19,982 from recurrent GBM lesions. Tumor cells. Endothelial cells. Immune cells.	TOP2A, MKI67, UBE2C, CENPF, PBK, VEGF.	Identified three separate cell types, from GBM lesions, from which 22 clusters were retrieved. Identified malignant cells in single-cell analysis using copy number variations. Identified high expression of proliferation-related genes in cell clusters. Detected VEGFA overexpression in almost all clusters.
Jain et al. [8] 2023	Serial trypsinization of 4385 GBM cells. Cancer-associated fibroblasts, epithelial cells, endothelial cells, and pericytes, immune cells.	ACTA2. COL1A1. TNC, S100A4. PDPN. PDGFRB.	Identified cancer-associated fibroblasts in GBM samples, and identified proximity to mesenchymal glioblastoma stem cells, endothelial cells, and M2 macrophages.
Zheng et al. [9] 2023	Fibroblasts. Chondrocytes Astrocytes. T_cells. Tissue_stem_cells. Monocyte.	571 genes related to necroptosis. ADORA2A. KDR. LAG3. EEF1B2. NDUFB2. RPL13. PTEN. EGFR. TTN.	A risk model was constructed using a Cox regression model with least absolute shrinkage and selection operator analysis, which included ten necroptosis-related genes.
LeBlanc et al. [10] 2022	>8000 single-cell genome, and >75,000 single-cell transcriptome profiles from 10 primary tumors and 2 recurrent tumors.	NRCAM. NCAM2. SHISA9 ACTA2. PDGFRB. VWF. MOG. MAG. ACTA2. PDGFRB.	Patient-derived explants (PDEs) can serve as a more accurate model for studying the complex heterogeneity of GBMs.
Yeo et al. [11] 2022	de novo mouse-made cells: 27,633 CD45- and 36,304 CD45+ cells. Dendritic cells (i.e., conventional or plasmacytoid), macrophages, T cells and natural killer cells, microglia, neutrophils, B cells, and mast cells. Distinct populations of EGFR+ cancer cells.	Upregulated pathways INFα/β/γ, cell migration, angiogenesis, oligodendrocyte differentiation, myelination and cell adhesion, and overexpression. Csfr3, Ccr1, Cxcr2, and Cxcr4 highly expressed in PMN-MDSCs.	Demonstrated relevant changes in the innate immune cell composition of the GBM microenvironment, with accumulation of myeloid-derived suppressor cells that promote immunosuppression.
Yesudhas et al. [12] 2022	3389 cells from four primary GBMs.	94 differentially expressed genes (DEGs) between tumor and periphery cells. CX3CR1, GAPDH, FN1, PDGFRA, HTRA1, ANXA2 THBS1, GFAP, PTN, TNC, VIM.	Insights into the heterogeneity of GBM and identifies novel disease-specific biomarkers, presenting potential avenues for the development of targeted therapies in GBM management.
Meng et al. [13] 2021	3589 cells from 4 cases.	DLL3. NEFL. NKX2-2. GABRA1. SOX2. SYT1. OLIG2. SLC12A5. FGFR3. ILR4. PDGFA. TRADD. EGFR. RELB. AKT2. CHI3L1(YKL40). NES. MET.	Reveals critical insights into intratumoral heterogeneity. This approach holds promise for improving the oncological management and outcomes of GBMs.
Chen et al. [14] 2021	17,132 cells from 50 cases. CD14 macrophages, CD3 T cells. SOX2 neuroglial cells.	499 genes in total. CSF1. CSF2. HGF. MCP-1. SDF-1. MFGE8. PDC001. PW039-705. PW035-710All. PJ052. PJ053.	MARCO macrophages found in GBMs correlate with worse prognosis. MARCO expression changes with anti-PD1 therapy. This indicates its potential as a biomarker for treatment response in GBM.
Xie et al. [15] 2021	Endothelial cells. Macrophages. Microglia. Neutrophils. T cells. B cells. Neuroglial cells. Vascular mural cells.	KLF2. TIMP3. SLC2A1. SLCO1A2. ABCG2. ABCB1. SLCO1A2. NET1. ATP10A. MYO1B. SPARC. ITGA5. PGF. NOTCH4. CD93. FABP1A. GNG11. SELE. VACM1. IL1B.	BBB transporters, including SLC2A1, ABCG2, ABCB1, SLCO1A2, and ATP10A, were elevated in endothelial cells, which impacts drug penetration and efficacy in brain tissue.
Mathewson et al. [16] 2021	8252 cells from 31 cases. T cells: CD8 T cells—CD4 conventional T cells—CD4 regulatory T cells—cycling T cells.	PRF1. GZMB. GZMA, GZMH. CLEC2D. NKG7. GNLY. KLRD1. FGFBP2. FCGR3A. S1PR5. KLRC1. KLRC3. KLRB1. KLRC2.	CLEC2D–CD161 pathway inhibition can enhance anti-tumor immune microenvironmental. NK-like receptor expression in GBM-infiltrating T cells implies that targeting these receptors could strengthen T-cell-based therapies.
Couturier et al. [17] 2020	53,586 glioblastoma cells. Glioblastoma stem cells.	TOP2A. FOXM1. USP1. APOD, OLIG2. SOX11. S100A10. HLA-4. APOE. HSPA1B.	Discovered a conserved trilineage hierarchy in glioblastoma centered around glial progenitor-like cells.
Liu et al. [18] 2020	3589 cells from 154 GBM patients in the TCGAGBM dataset and 155 GBM patients in the GSE16011 dataset.	FERMT1. COL22A1. LOXL1. PCDHB3. TCAF2. HOXB2. HOXD11, PTPRN. TSHZ2.	Prognostic model that incorporated factors such as radiotherapy status, and age to predict survival probabilities, suggesting that these genes could serve as potential prognostic biomarkers.
Neftel et al. [19] 2019	7930 cells from 28 cases. Macrophages. Oligodendrocytes. T cells. Astrocytes	5730 genes in total. HILPDA. DDIT3. ENO2 and LDHA. MGH125. MGH102. EGFR. PDGFRA. CDK4.	High-level amplifications of EGFR, PDGFRA, and CDK4 influence cellular states within the GBM microenvironment. PDGFRA and CDK4 amplifications correlate with the expansion of NPC and OPC, respectively.
Darmanis et al. [20] 2017	3589 cells from 4 cases. Tumor cells. Vascular cells. Oligodendrocytes. OPCs. Neurons. Astrocytes.	MBP. OPALIN. GPR17. L1CAM. ALDH1L1. WIF1. NTSR2. PECAM-1. NFIB. SOX9. Higher expression of hypoxia and adhesion-related genes in the tumor core.	Identified infiltrating neoplastic cells in peripheral regions of the core lesions, representing intratumor heterogeneity. Identified consistent gene signature between patients. Identified myeloid cell populations in the tumor core and surrounding peritumoral space.
Patel et al. [21] 2013	430 cells from 5 cases. NPC. Neurons. Mesenchymal cells.	EGFR. PDGFRA. PDFGA. FGFR1. FGF1. NOTCH2. JAG1.	Identified intratumor heterogeneity by identifying different GBM subtypes within the tumors. High tumor heterogeneity was associated with poor prognosis.
Müller et al. [22] 2017	672 cells identified. Tumor-associated macrophages (TAMs) from 5 GBMs.	Upregulated genes in blood-derived TAMs include those of immunosuppressive cytokines (specific genes not mentioned).	Blood-derived TAMs infiltrate pretreatment GBMs and exhibit immunosuppressive characteristics, presenting a barrier to immunotherapy.
Little et al. [23] 2012	41,997 cells were counted across 190 distinct loci.	EGFR (upregulated), PDGFRA (upregulated).	Intratumoral heterogeneity in glioblastoma complicates treatment strategies, as different cell populations with distinct gene amplifications may contribute variably to disease progression and response to therapies.
Lai et al. [24] 2022	2305 cancer cells from tumor cores.	LITAF (Downregulated), MTHFD2 (Upregulated), NRXN3 (Upregulated), OSMR (Upregulated), RUFY2.	Novel prognostic model for predicting survival in GBM patients by integrating scRNA-seq and bulk RNA-seq datasets.
Yu et al. [25] 2020	6148 cells identified (from 7928 single-cell transcriptomes).	EGFR (Upregulated) cells, PTPRZ1 (Upregulated), SOX2 (Upregulated), MKI67 (Marker for proliferation), HYDIN, FOXJ1.	scRNA-seq can uncover distinct cellular states and gene expression profiles that are critical for understanding tumor progression and therapeutic resistance in GBM. Emphasis made on the importance of multi-sector biopsies to capture the heterogeneity of gliomas effectively.
Lemée et al. [26] 2015	Not specified.	Genes related to stem cell phenotype: CD133, Sox2, nestin, musashi 1 (upregulated). Invasion-related genes: Galectin-1, Rac1, Rac3, RhoA GTPases, p27, avb3 integrin (upregulated). Cell adhesion-related genes: CDH20, PCDH19 (upregulated). Migration-related genes: SNAI2, NANOG, USP6, DISC1 (upregulated). Immune response: TLR4 (upregulated). Angiogenesis: HEG1, VEGFR2 (upregulated).	Emphasis made on the importance of understanding the peritumoral brain zone (PBZ) in GBM, highlighting that it contains tumor and stromal cells that promote growth and invasion.
Lee et al. [27] 2017	305 single cells from 7 samples of 3 patients.	EGFR, PIK3CA.	Different single cells exhibited various EGFR alterations, indicating late events in tumor evolution. The presence of transcriptional heterogeneity suggests that 5-ALA (-) tumors can still harbor aggressive tumor markers despite being perceived as being less aggressive.
Pine et al. [28] 2020	62,885 cells identified. Neural progenitor-like cells (NPC-like), Oligodendrocyte progenitor-like cells (OPC-like), Astrocyte-like cells (AC-like), Mesenchymal-like cells (MES-like).	SOX4 (upregulated), BCAN (upregulated and associated with invasiveness), DLL3 (upregulated), KPNA2 (upregulated and promotes metabolic reprogramming).	Compared scRNA-seq across four patient-derived glioblastoma stem cell models, including glioma spheres, brain organoids, glioblastoma cerebral organoids, and patient-derived xenografts. Successfully recapitulated cellular states commonly found in primary tumors.
Sullivan et al. [29] 2014	Not specified.	SERPINE1, TGFB1, TGFBR2, and VIM (all upregulated). ASCL1, GFAP, NCAM1, and SOX9 (all downregulated), TWIST1, and NF-kB. EGFR amplification.	Circulating tumor cells exhibit higher mesenchymal and lower neural differentiation, contributing to invasiveness and possibly rare metastases.
Jacob et al. [30] 2020	scRNA-seq data from organoids derived from 53 patient cases and established 70 glioblastoma organoid (GBO) samples.	EGFR (including variant III—EGFRvIII), SOX2, and NESTIN.	Organoids retained transcriptomic signatures, cell-type diversity, and molecular properties of parental tumors.

NPC = neural progenitor cell; OPC = oligodendrocyte progenitor cells; MARCO = macrophage marker in GBM; and GBO = glioblastoma organoid.

**Table 2:** Current roles of scRNA-seq in glioblastomas.

Potential Diagnostic
- Genetic factors of tumor cells, endothelial cells, fibroblasts, and various immune cells; - Single-cell genetic factors that shape the composition of GBM neoplastic cells; - Characterize the coexistence of GBM different genetic tumoral cells; - Cells from the same tumor tissue can exhibit different mutations, leading to various phenotypic and epigenetic changes; - Characterize the spatiotemporal cellular genomic architecture of GBM; - Novel tumor antigens based on single-cell genetics; - Understand and target genetic drivers of tumor recurrence; - Define the genetics of immunosuppressive cell populations; - Describe the genetic tumor mechanisms of immune escape in GBM; - Mitochondrial subtype with therapeutic vulnerabilities.
**Therapeutic**
- Modulate drug delivery based on BBB cellular genetic phenotype; - Use differentiation therapy towards stem-like cells to arrest tumor growth; - Target the immunosuppressive TAMs; - Combinatorial therapeutic strategies to target all tumor areas and cells; - Withdrawal of ineffective treatments to prevent side effects and toxicity; - Oncological cell treatments that target pro-tumoral factors; - Design second-line immunotherapy drugs.
**Prognostic role of scRNA-seq in GBM**
- Immunotherapy-resistant oncological cell subsets; - Identify and target drivers of tumor recurrence; - Novel tumor mechanisms of immune escape.

GBM = glioblastoma; TAM = tumor-associated macrophages; scRNA-seq = single-cell RNA sequencing; BBB = blood–brain barrier.

## Abbreviations

BBB = blood–brain barrier; NGS = next-generation sequencing; CNS = central nervous system; scRNA-seq = single-cell RNA sequencing; TME = tumor microenvironment; GBM = glioblastoma; ncRNAs = non-coding RNAs; qRT-PCR = quantitative reverse-transcription polymerase chain reaction; cDNA = complementary DNA; MDSCs = myeloid-derived suppressor cells.
